# Genome-Wide Profiling of H3K56 Acetylation and Transcription Factor Binding Sites in Human Adipocytes

**DOI:** 10.1371/journal.pone.0019778

**Published:** 2011-06-02

**Authors:** Kinyui Alice Lo, Mary K. Bauchmann, Amy P. Baumann, Christopher J. Donahue, Mark A. Thiede, Lisa S. Hayes, Shelley Ann G. des Etages, Ernest Fraenkel

**Affiliations:** 1 Department of Biological Engineering, Massachusetts Institute of Technology, Cambridge, Massachusetts, United States of America; 2 Computer Science and Artificial Intelligence Laboratory, Massachusetts Institute of Technology, Cambridge, Massachusetts, United States of America; 3 Genetically Modified Models Research Center of Emphasis, Pfizer Global Research and Development, Groton, Connecticut, United States of America; 4 Cardiovascular, Metabolic and Endocrine Diseases, Pfizer Global Research and Development, Groton, Connecticut, United States of America; University of Frankfurt-University Hospital Frankfurt, Germany

## Abstract

The growing epidemic of obesity and metabolic diseases calls for a better understanding of adipocyte biology. The regulation of transcription in adipocytes is particularly important, as it is a target for several therapeutic approaches. Transcriptional outcomes are influenced by both histone modifications and transcription factor binding. Although the epigenetic states and binding sites of several important transcription factors have been profiled in the mouse 3T3-L1 cell line, such data are lacking in human adipocytes. In this study, we identified H3K56 acetylation sites in human adipocytes derived from mesenchymal stem cells. H3K56 is acetylated by CBP and p300, and deacetylated by SIRT1, all are proteins with important roles in diabetes and insulin signaling. We found that while almost half of the genome shows signs of H3K56 acetylation, the highest level of H3K56 acetylation is associated with transcription factors and proteins in the adipokine signaling and Type II Diabetes pathways. In order to discover the transcription factors that recruit acetyltransferases and deacetylases to sites of H3K56 acetylation, we analyzed DNA sequences near H3K56 acetylated regions and found that the E2F recognition sequence was enriched. Using chromatin immunoprecipitation followed by high-throughput sequencing, we confirmed that genes bound by E2F4, as well as those by HSF-1 and C/EBPα, have higher than expected levels of H3K56 acetylation, and that the transcription factor binding sites and acetylation sites are often adjacent but rarely overlap. We also discovered a significant difference between bound targets of C/EBPα in 3T3-L1 and human adipocytes, highlighting the need to construct species-specific epigenetic and transcription factor binding site maps. This is the first genome-wide profile of H3K56 acetylation, E2F4, C/EBPα and HSF-1 binding in human adipocytes, and will serve as an important resource for better understanding adipocyte transcriptional regulation.

## Introduction

White adipose tissue is a key organ in the regulation of whole body energy homeostasis [1]. Besides acting as a passive fat storage organ, it serves to regulate systemic energy homeostasis by secreting various soluble mediators, widely termed adipokines, examples of which include leptin, adiponectin, visfatin and resistin [2]. It has been recognized that an increase in white adipose tissue mass is associated with insulin resistance, a major cause of diabetes, hypertension and cardiovascular diseases. With the growing epidemic of obesity, a better understanding of adipocyte biology could potentially lead to novel drug targets to tackle obesity-related metabolic diseases.

Transcriptional controls have emerged as a particularly important aspect of cellular regulation that can be exploited for therapeutic benefit in diabetes. Both the most widely prescribed anti-diabetic treatment, metformin, as well as the more recently developed thiazolidinediones act by modulating the activity of transcriptional regulatory proteins [3], [4]. Recent advances in high-throughput technologies now provide methods for examining transcriptional regulation across the genome, potentially revealing key transcriptional regulators that could serve as potential drug targets. For example, chromatin immunoprecipitation followed by microarray analysis (ChIP-chip) or next generation massively parallel sequencing (ChIP-seq) can be used to probe genome-wide DNA-protein interactions. Currently, most of the ChIP-chip or ChIP-seq experiments on adipocytes have been done in cell lines of murine origin such as the preadipocyte cell line 3T3-L1 [5], [6], [7]. Given that tissue-specific transcription has diverged significantly between human and mouse [8], there is a need to construct a human adipocyte transcriptional regulatory network.

Changes in histone acetylation appear to have a central role in insulin function and insulin resistance. Both insulin and metformin have been shown to alter the activity of the histone acetyltransferases (HATs), CBP and p300 [9]. In addition, although CBP heterozygous mice are lipodystrophic, they are protected from diet-induced insulin resistance [10]. Furthermore, the histone deacetylase (HDAC) SIRT1, by virtue of its role in inflammatory responses, adipokine secretion and its interaction with the insulin signaling pathway, is suggested to have therapeutic benefit in treating insulin resistance [11]. Recently, it has been shown that the lysine 56 of histone H3 (H3K56) is acetylated by CBP and p300 and deacetylated by SIRT1 and SIRT2 [12]. Given the roles that HATs and HDACs play in controlling insulin sensitivity, and that the H3K56 acetylation status is a consequence of the combined activity of HATs and HDACs, we measured the genome-wide distribution of H3K56 acetylation in normal human adipocytes.

The presence of H3K56 acetylation has only been recently discovered in humans [12], therefore the molecular function and significance of H3K56 acetylation in humans is less studied than in yeast. It has been shown that in human embryonic stem cells prior to differentiation, H3K56 acetylation marks targets of the key regulators of pluripotency, namely NANOG, SOX2 and OCT4 [13]. Upon cellular differentiation, H3K56 acetylation relocates to developmental genes.

In this study, we measured sites of H3K56 acetylation across the genome in mature adipocytes derived from human mesenchymal stem cells (hMSC) [14], which provide a good *in vitro* model to study protein-DNA interaction in human adipocytes and complement the more widely used mouse cell lines 3T3-L1 and 3T3-F442A. We showed that in these cells, H3K56 acetylation is associated with almost half of the genome and it primarily occurs around transcription start sites. Genes with this modification were generally associated with higher expression, and genes with the highest levels of H3K56 acetylation are enriched for transcription factors, adipokine signaling and diabetes-related proteins. We used computational methods to identify DNA-binding proteins that might recruit acetyltransferases and deacetylases to the sites of H3K56 acetylation. We found a strong enrichment of the E2F motif in these regions and demonstrated using ChIP-seq that E2F4 binds many of these loci. ChIP-seq analysis also showed that the majority of HSF-1- and C/EBPα-bound genes are enriched for H3K56 acetylation. At sites where the H3K56 acetylation modification and transcription factor binding co-occur, H3K56 acetylation is usually adjacent to but not directly overlapping the transcription factor binding sites. These data provide the first genome-wide view of H3K56 acetylation in human adipocytes, paving the way for future studies aimed at understanding how epigenomic modifications influence obesity-related diseases.

## Results

### Differentiation of mature human adipocytes

**Figure 1:**
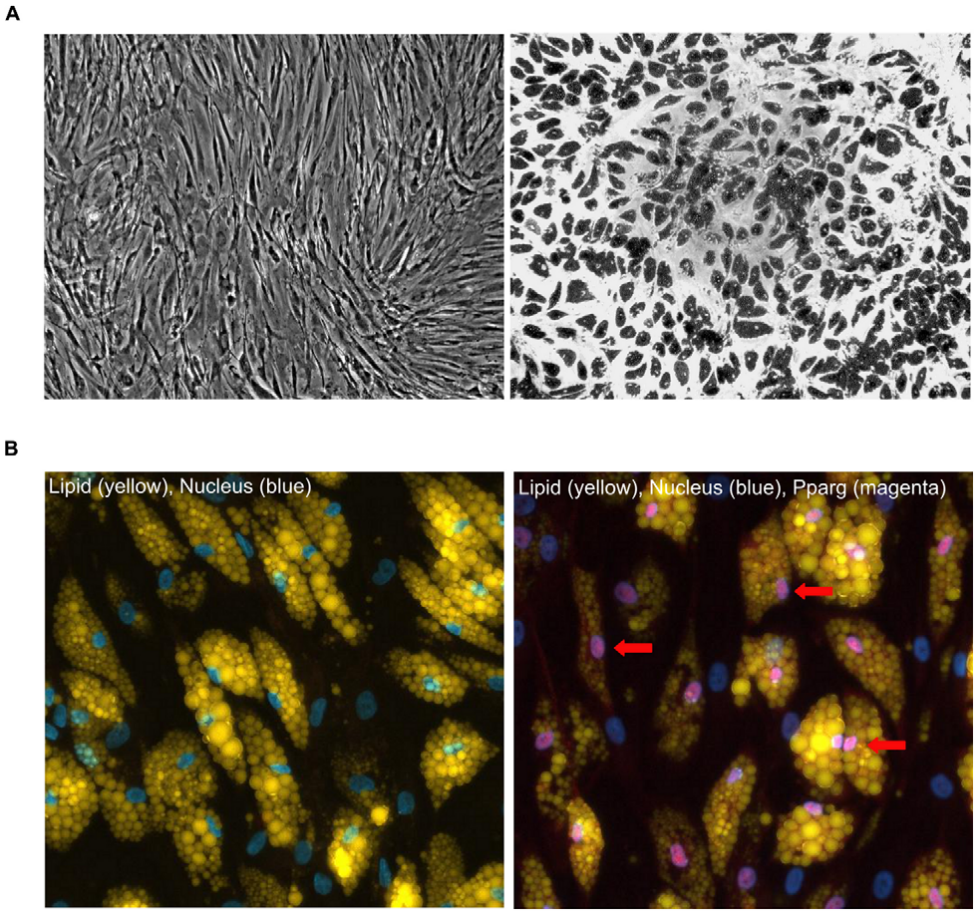
Images of bone marrow mesenchymal stem cells before and after adipogenic differentaion. (A) The left panel shows 4x phase contrast microscope images of undifferentiated human bone marrow mesenchymal stem cells and the right panel shows the differentiated adipocytes at day 19. (B) Cellomics image analysis of the human bone marrow mesenchymal stem cells-derived adipocytes after 19 days of differentiation, fixed and stained with DAPI (nuclei-blue), Nile red (a selective fluorescent stain for intracellular lipid droplets-yellow), and specific antibodies for PPARγ (Alexa 647-magenta). The left panel shows the lipid and nuclei staining and the right panel has additional PPARγ staining, three of which are indicated with red arrows.

Since we are interested in studying transcriptional regulation in human adipocytes, we derived mature adipocytes from human bone marrow mesenchymal stem cells purchased from Lonza. Differentiation took 19 days, including 14 days in adipogenic differentiation media and 5 days in adipogenic maintenance media (see Methods for details). Figure 1A shows that before differentiation, cells exhibit an elongated and fibroblast-like morphology. After differentiation, cells become more round and accumulate multiple intracellular lipid droplets. Using the Cellomics high content imaging system, we determined the differentiation efficiency of replicate samples of 6 independent differentiation experiments (Table S1) with cells from different passages (only cells less than passage 8 were used). We estimated that the differentiation efficiency was 61% ±4 %, as determined by the number of cells that have intracellular lipid droplets (stained by Nile red) over the total number of nuclei observed (stained by DAPI). Cellomics data also show that about 54% of the cells were positive for the adipocyte marker, PPARγ, and that the PPARγ staining largely overlaps with that of DAPI (Figure 1B), as expected for mature adipocytes. We developed a purification process to separate mature adipocytes, which are buoyant during centrifugation, from immature adipocytes, which pellet. Based on visual examination under the microscope, our adipocyte preparation appears to be at least 90% mature adipocytes.

### Overall analysis of H3K56 acetylation sites

After crosslinking the mature adipocytes using formaldehyde which captures *in vivo* protein-DNA interactions, we performed chromatin immunoprecipitation with an antibody that specifically targets the acetylated lysine 56 of histone 3. The specificity of the antibody for this particular histone acetylation site has been previously shown in ChIP experiments and other applications [12], [15], [16]. Immunoprecipitated protein-DNA complexes were reverse-crosslinked, purified DNA was sequenced using the Illumina Genome Analyzer II, and the resulting sequences were used to identify enriched regions as described in Materials and Methods. A gene was considered to have H3K56 acetylation if there was at least one ChIP-seq peak within 10 kilobases of the transcription start site. The 10-kilobase window was chosen in light of a recent report showing that a regulatory site's influence on expression falls off almost linearly with distance from the nearest transcription start site in a 10-kilobase range [17]. The number of reads, peaks and genes bound/enriched for each ChIP-seq experiment performed is listed in Table S3.

**Figure 2:**
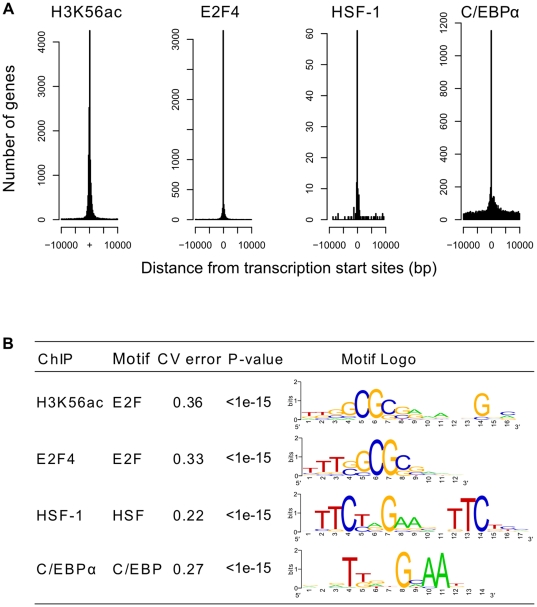
Genome-wide analysis of H3K56 acetylation and transcription factor binding in human adipocytes. (A) Histograms showing that the distributions of peaks for all four ChIP-seq experiments are centered on transcription start sites; the X-axis represents distance from transcription start sites and the Y-axis represents frequency. H3K56ac is H3K56 acetylation (B) Sequence motifs associated with the enriched regions in each ChIP-seq experiment. Only the highest ranked motif is shown. Cross-validation errors (“CV error”) and p-values were calculated as described in Methods.

Using these criteria, we found that 10,215 genes have H3K56 acetylation. While the majority of the H3K56 acetylation sites occur at the proximal promoter region (Figure 2A), there is a drop in H3K56 acetylation right before transcription start sites (Figure S1). The same observation has been made on genome-wide data for other histone modifications such as H3K4me2 and H3K4me3 in hematopoietic cells [18]. These findings suggest that there is a depletion of nucleosomes immediately upstream to transcription start sites. 

### Genes enriched for H3K56 acetylation are also targets of three different transcription factors

In order to discover the DNA-binding proteins that recruit acetyltransferases and deacetylases to sites of H3K56 acetylation, we searched for the DNA sequence motif associated with the H3K56 acetylation-enriched regions using the THEME algorithm [19]. The top motif obtained was that of E2F (Figure 2B), suggesting that members of this family of proteins might bind to these loci. To verify this, we performed ChIP-seq on the cell cycle regulator E2F4. E2F4 was found to bind to 5,340 genes, of which a very high proportion (87%) was also enriched for H3K56 acetylation. We not only found that H3K56 acetylation and E2F4 binding occur at a similar set of genes, we also found that the acetylated and binding locations are usually close to each other, with 74% of E2F4 binding sites occurring within 300 base pairs of an H3K56 acetylation site (Table S4). The genomic distribution of E2F4 was highly similar to that of H3K56 acetylation (Figure 2A), with the majority of the sites proximal to transcription start sites. The frequent association of E2F4 binding and H3K56 acetylation supports the hypothesis that E2F4 recruits HATs and HDACs to these sites.

HATs and HDACs also modify DNA binding proteins in addition to acetylating and deacetylating histones respectively. It is interesting that three of the proteins known to alter acetylation of H3K56, namely CBP, p300 and SIRT1, are also known to acetylate and deacetylate the master regulator of heat shock response, HSF-1, affecting its DNA binding activity and expression of its target genes [20]. In order to determine if HSF-1 and H3K56 acetylation share similar target genes, we performed ChIP-seq on HSF-1. Our genome-wide HSF-1 data show that in mature human adipocytes, HSF-1 binds to 174 genes, 90% are also enriched with H3K56 acetylation and 69% of the peaks associated with HSF-1-bound genes are within 300 base pairs of an H3K56 acetylation site.

E2F4 and HSF-1 are ubiquitously expressed transcription factors, whose functions appear to be conserved across many tissues, raising the question as to whether H3K56 acetylation might also be associated with the binding of adipocyte-specific proteins. To explore this question, we profiled the genome-wide binding sites of the master regulator of adipocytes, C/EBPα. C/EBPα binds to 5,818 genes, of which 74% also have an H3K56 acetylation mark. The complete list of genes that have H3K56 acetylation and are also bound by at least one transcription factor is listed in Table S5.

In order to verify the acetylation and transcription factor binding sites identified by the above four ChIP-seq experiments, we selected 21 positive regions and 2 negative regions to perform independent ChIP-qPCR experiments (Figure S2) in biological triplicates. We found that among the 21 positive loci tested, 20 show significant enrichment when compared to the IgG control. The 2 negative control regions show no enrichment in all the ChIP-qPCR experiments performed.

When we examined the percent of overlap between H3K56 acetylation sites and the binding sites of the different transcription factors, we noticed that while HSF-1 and E2F4 are very often recruited to regions where H3K56 acetylation occurs, this is not as common for C/EBPα: only 31% of the peaks associated with the C/EBPα-bound genes are within 300 base pairs of an H3K56 acetylation site. Furthermore, in contrast to the enriched regions of E2F4 and H3K56 acetylation, C/EBPα-enriched regions have a more dispersed distribution throughout the 10-kilobase window around transcription start sites (Figure 2A), and a large proportion of C/EBPα peaks (79%) lies beyond 10 kilobases from transcription start sites. The more widespread distribution of C/EBPα binding sites agrees with the study by Lefterova *et al* [6]. When we compared our C/EBPα data set with a recently published PPARγ ChIP-seq data set in human adipose stromal cells derived adipocytes [21] , 43% of the C/EBPα-enriched regions (14,174 out of 32,864) overlapped with the PPARγ-enriched regions, while a previous study comparing C/EBPα and PPARγ ChIP-chip data in 3T3-L1 [6] using promoter arrays shows that the two factors share 63% of their binding sites. The lower percentage of overlap in human adipocytes could be due to species differences and we are comparing C/EBPα and PPARγ binding sites in two different human adipocyte systems; it could also be because the ChIP-seq technology recovers many more distal binding sites.

### Bound genes are associated with higher expression

**Figure 3:**
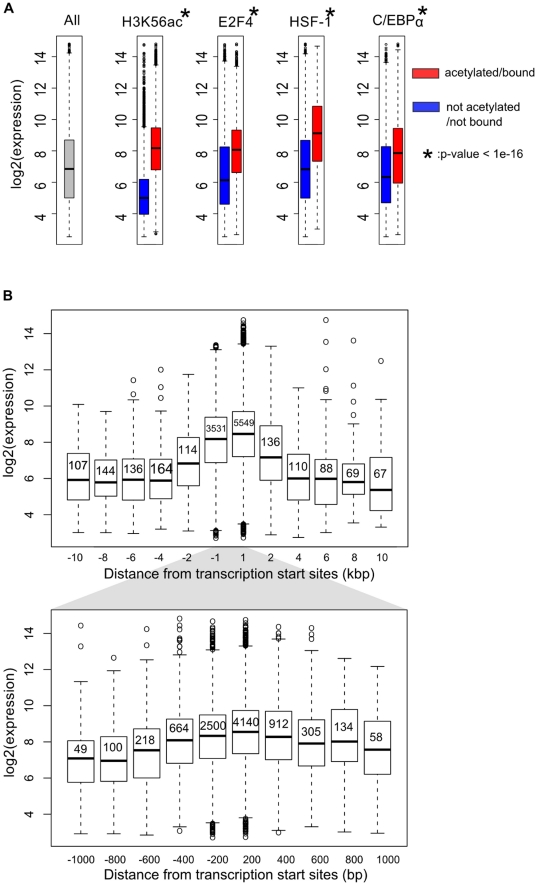
Genes that are bound by transcription factor or acetylated at H3K56 are associated with higher expression. (A) Comparison of the expression level of all genes (left panel) to genes that have (red) or do not have (blue) H3K56 acetylation or transcription factor binding sites. The dark band in the middle of the each box represents the median expression value of all gene that have/do not have an H3K56 acetylation or transcription factor binding sites. Asterisks represent statistical significance with p-values<1e-16. (B) The top panel shows the distribution of expression values for genes that have been categorized based on the distance from their transcription start sites to the nearest H3K56 acetylation peak. The number within each box represents the number of genes in each category. The bottom panel is similar to the top one, zooming in around -1000 to +1000 base pair window.

Histone acetylation is generally found to be associated with gene activation, while particular DNA-binding proteins can either activate or repress transcription. We compared our data to gene expression data from mature human adipocytes (Des Etages, S. *et al.* manuscript in preparation) and found that genes with H3K56 acetylation are associated with higher expression (Figure 3A). However, similar to what has been observed in human embryonic stem cells [13], H3K56 acetylation marks are not restricted to highly expressed genes; some unexpressed or poorly-expressed genes also harbor this modification, suggesting that H3K56 acetylation is not simply a marker for gene activity. We divided genes that have H3K56 acetylation based on the distance from their transcription start sites to the nearest acetylation site. We found that genes with one or more H3K56 acetylation sites within 2 kilobases of transcription start sites have significantly higher level of expression compared to the unmodified genes or genes with the modification further away from transcription start sites (p-value<1e-15; Figure 3B). Similarly, genes that are bound by E2F4, HSF-1 or C/EBPα are also associated with higher expression (Figure 3A). The differences of the expression distributions between bound and unbound genes for all the transcription factors under study are highly significant (p-value<1e-15).

### Many genes for adipocyte transcription factors are highly acetylated at H3K56

**Figure 4:**
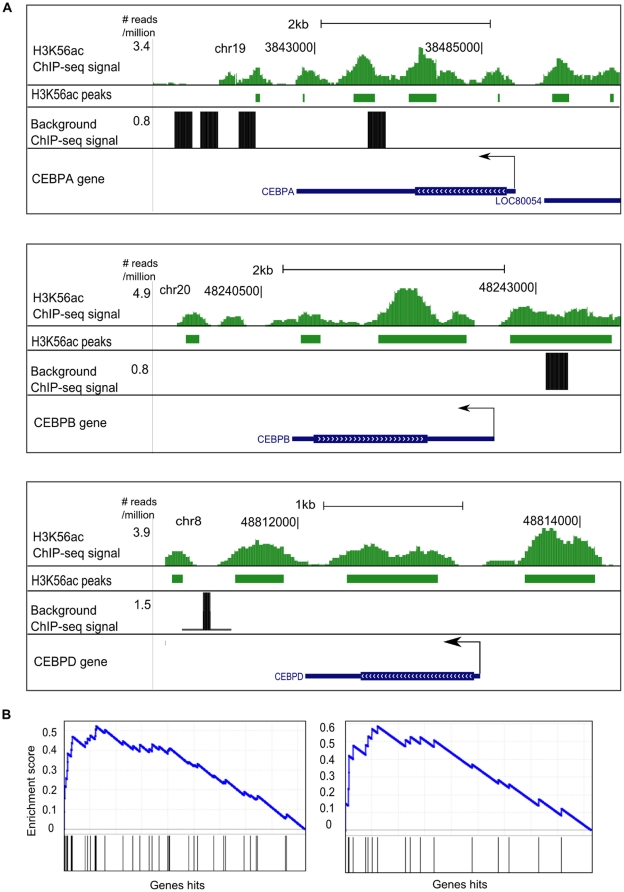
Genomic maps of examples of H3K56 acetylation-enriched genes. (A) H3K56 acetylation modifications were found on the promoters and gene body of C/EBPα, C/EBPβ and C/EBPδ. Background represents the mock IgG experiment reads. Rectangular boxes below the H3K56 acetylation peaks represent the enriched regions. The Y-axis represents the number of reads per million sequenced, note that each ChIP experiment was plotted on a different scale on the Y-axis. Arrows indicate the direction of transcription. (B) Profiles from gene set enrichment analysis. Left: HSA4920 Adipocytokine signaling pathway. Right: HSA04930 Type II Diabetes Mellitus. Genes were ranked based on the number of acetylated sites per gene. Black lines represent genes with the specified annotation. The Y-axis represents the enrichment score (see [23]).

Our genome-wide H3K56 acetylation data reveal that a surprisingly large number of genes (10,215) are enriched for this histone modification. We hypothesized that genes with very high levels of acetylation might be functionally distinct from those with lower levels of acetylation. Indeed, when we ranked the genes with H3K56 acetylation by the number of acetylated sites per gene, we found that 47 of the top 161 genes (all of which have five or more H3K56 acetylation sites) are annotated as transcription factors, (p-value: 3.5e-11) and twelve of them are from the homeobox structural class (EMX2, HOXA10, HOXA9, HOXB2, HOXC9, IRX5, LMO4, MEIS1, MEIS2, PRRX1, SATB2 and SIX2; p-value: 4.8e-7). Many homeobox transcription factors are involved in the control of gene expression during morphogenesis and development, and some of them have been shown to have important roles in the differentiation of adipocytes [22]. This observation is in agreement with the observation that developmental genes are hyperacetylated at H3K56 in differentiated cells [13]. Other important adipocyte-related transcription factor genes that are hyperacetylated include CEBPA, CEBPB, CEBPD, KLF6, KLF9, FOXC1, IRF1, PPARA, RXRA and THRA (Figure 4A).

To search more systematically for classes of genes with high levels of H3K56 acetylation, we ranked the 10,215 genes with H3K56 acetylation by the number of H3K56 acetylation sites per gene. Gene set enrichment analysis [23] identified a number of gene sets that were overrepresented at the top of this list, including the KEGG adipokine signaling pathway and the KEGG type II Diabetes Mellitus (T2DM) pathway (Figure 4B). Thus, high levels of H3K56 acetylation may be associated with regulation of adipokine secretion and the etiology of T2DM. All the gene sets that have a family-wise error rate (FWER) of less than 0.05 are listed in Table S7.

### C/EBPα-bound targets are enriched for lipid metabolic genes and adipokines

The genome-wide binding data of C/EBPα recovered many known regulatory sites on genes that are important in adipogenesis and adipocyte function, including FABP4/aP2, a fatty acid binding protein; CD36, a regulator of fatty acid transport; LIPE/HSL, an enzyme that regulates hormone-mediated lipolysis; OLR1, a low density lipoprotein receptor, and ME1, an enzyme that generates NADPH for fatty acid biosynthesis. The top gene ontology (GO) category that is associated with the C/EBPα-bound genes is lipid, fatty acid and steroid metabolism (p-value: 4.3e-4, Table S8). As previously reported, C/EBPα regulates transcription of the human adiponectin gene through an intronic enhancer [24]. We were able to confirm the existence of such an enhancer in our model of hMSC-derived adipocytes (Figure S3). In addition, we also observed C/EBPα binding sites on the resistin promoter and retinol-binding protein 4 (RBP4) promoter, suggesting that C/EBPα may have a role in mediating the expression of adipokines.

The human C/EBPα gene is almost 90% homologous to its rat and mouse counterparts [25]. Because of this high degree of homology, we asked if the majority of the human C/EBPα-bound genes are also bound in mouse. To determine the degree of conservation of C/EBPα binding between human and mouse adipocytes, we compared our C/EBPα ChIP-seq data with the published C/EBPα ChIP-chip data in 3T3-L1 [6]. There are 3,998 C/EBPα-bound genes in 3T3-L1, out of which 3,345 have human orthologues. We found that 48% (1,636 out of 3,345) of the C/EBPα targets in 3T3-L1 are also targets in human adipocytes. Most (72%, 4,182 out of 5,818) of the human adipocyte C/EBPα target genes are unique. Some of the differences between these data sets are undoubtedly due to the fact that they were collected with different experimental techniques. However, the data are also consistent with the observations that binding sites have diverged significantly between human and mouse for several other highly conserved transcription factors [8].

### HSF-1 binds to classic heat shock protein genes in unstimulated human adipocytes

HSF-1 is a transcription factor induced by diverse environmental and physical stresses. Our experiment provides the first comprehensive view about the role of HSF-1 in human adipocytes. The genome-wide HSF-1 data show that in mature adipocytes, HSF-1 binds to 174 genes, examples of which are DNAJB6, HSPH1, HSPA9, HSPD1, HSPB1, CCT4 and CCT7; many of the bound genes are involved in protein folding (p-value: 8.4e-17), protein metabolism and modification (p-value: 2.2e-8), protein complex assembly (p-value: 2.9e-5) and stress response (p-value: 2.8e-4) (Table S8). This finding highlights the fact that even in unstimulated and unstressed adipocytes, HSF-1 binds DNA. This is the first time that HSF-1 binding sites have been profiled in human adipocytes. It will be interesting to compare HSF-1 binding sites in other quiescent tissues under non-heat shock conditions.

### E2F4 targets in human adipocyte are highly enriched for cell cycle genes

We found that in human adipocytes, E2F4 binds to many genes (5,340) that have not previously been recognized as its targets; the majority of the binding sites are located proximal to transcription start sites: 83.5% and 61.9% are located less than 1000 and 100 bases from transcription start sites, respectively. The top GO categories associated with the E2F4-bound genes are DNA metabolism (p-value: 5.1e-14), cell cycle (p-value: 1.1e-15) and chromatin packaging and remodeling (p-value: 5.0e-9) (Table S8). True to its role as a cell cycle regulator, E2F4 binds to many of the well-known cyclin-dependent kinases (CDK2, CDK4, CDK5) and cyclin-dependent kinase inhibitors (CDKN1A, CDKN1B, CDKN1C, CDKN2A, CDKN2C, CDKN2D and CDKN3) in human adipocytes.

In order to assess the differences in binding among different human tissues, we compared our E2F4 ChIP-seq data in adipocytes with the published E2F4 ChIP-chip data in human liver, pancreatic islets, pancreatic acinar tissues and the liver carcinoma cell line HepG2 [26]. Using promoter arrays capturing up to 1 kilobase immediately upstream of most transcription start sites, Conboy *et al.* found that there are 441 genes common among the four human tissues/cell types. We found that among these 441 genes, almost half (195 genes) were bound by E2F4 in adipocytes. A large proportion of the common genes were related to the cell cycle process (60 out of 199, p-value: 5.02e-14), in accord with the previous finding that cell cycle genes are the conserved targets of E2F4 [26].

### Transcription factor binding sites and H3K56 acetylation marks are often adjacent but do not overlap

**Figure 5:**
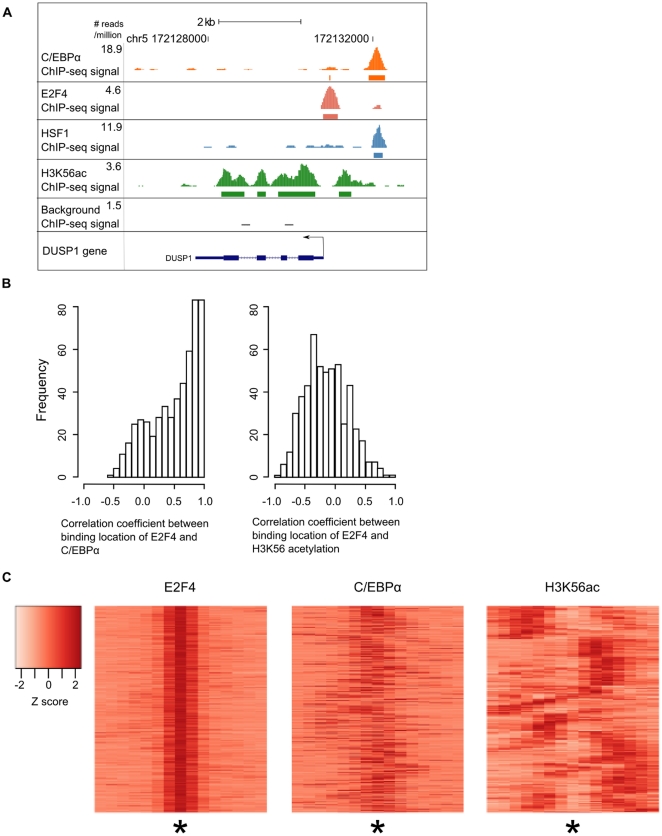
Correlation in binding locations of C/EBPα, E2F4 and H3K56 acetylation. (A) DUSP1 is bound by multiple transcription factors and also has multiple H3K56 acetylation enrichment regions. The rectangular boxes below the peaks represent the enriched regions. (B) Histogram showing the correlation coefficients representing similarity between the relative locations of C/EBPα and E2F4 enrichment signal (left) and E2F4 and H3K56 acetylation enrichment signal (right). (C) Heatmaps showing the intensity of bound regions centered on E2F4 peaks, indicated by an asterisk. Each row in the heatmap represents each binding region, which has been divided into fifteen bins of one hundred base pairs. The color scale indicates the Z score of the number of reads in each bin. The immunoprecipitated proteins are E2F4 (left), C/EBPα (middle) and H3K56 acetylation (right).

Comparing the genome-wide H3K56 acetylation distribution with the binding sites of C/EBPα, HSF-1 and E2F4, we found that the majority of the genes that are bound by each protein are also acetylated at H3K56 (p-value<1e-15, Table S4). The fraction of the transcription factor binding sites that is within 300 base pairs of a site of acetylation ranges from 31% to 74%. Despite the proximity of these sites, we found that transcription factor binding sites rarely occur at exactly the same location as the strongest sites of acetylation. An example is illustrated by the DUSP1 gene, which has multiple H3K56 acetylation sites at the promoter and gene body (Figure 5A). It also has C/EBPα, E2F4 and HSF-1 binding sites within 2 kilobases from the promoter. Figure 5A shows that C/EBPα and HSF-1 bind at the same genomic location, and E2F4 binds closer to the transcription start site. However, none of the binding sites of the above three transcription factors overlap with the H3K56 acetylation regions. On the genome-wide level, we examined genes that have H3K56 acetylation as well as E2F4 and C/EBPα binding sites within 1500 base pairs of each other (we excluded the HSF-1 data as HSF-1 only binds to less than 200 genes genome-wide). For each region, we computed the pairwise correlation coefficient of binding location between transcription factors and between each transcription factor and H3K56 acetylation (see Methods). E2F4 and C/EBPα binding locations often show positive correlation, and a significant number even have a perfect correlation with r = 1, suggesting that the binding sites directly overlap one another. On the other hand, E2F4 and H3K56 acetylation rarely exhibit perfect correlation and they are often negatively correlated (Figure 5B). We display the ChIP-seq enrichment signal across 1500 base pairs, center around the strongest E2F4 peak in a heatmap format (Figure 5C). The heatmap clearly illustrates that binding of E2F4 and C/EBPα often occur at identical genomic loci, but this is not the case for E2F4 and H3K56 acetylation.

## Discussion

The binding of transcription factors and the modification of histones are highly interdependent processes that together determine the epigenetic state of the cell and regulate gene expression. A more thorough enumeration of genome-wide transcription factor binding sites and histone modifications, combined with gene expression analysis, could lead to a better understanding of how gene regulation occurs. This systems approach could be of great use in identifying better mechanisms for manipulating transcription for therapeutic purposes.

We have presented the genome-wide locations for the acetylation of H3K56, a histone modification regulated by CBP, p300 and SIRT1, all of which have been implicated in insulin resistance. To better understand the mechanism by which these histone modifying enzymes are recruited to these loci, we used sequence analysis to discover motifs that are overrepresented near sites of H3K56 acetylation. We observed enrichment for the binding sequence for E2F proteins and used ChIP-seq to confirm a dramatic enrichment of E2F4 bound genes at H3K56 acetylation regions. Surprisingly, we found that the binding sites of two other transcription factors, HSF-1 and C/EBPα, also show a significant degree of overlap with the sites of H3K56 acetylation (Table S4). Approximately 70% of the binding sites for each of HSF-1 and E2F4 and approximately 30% of the C/EBPα sites are within three hundred bases of a site of H3K56 acetylation. The very high frequency of H3K56 acetylation near HSF-1 and E2F4 raises the interesting possibility that these two proteins may recruit the acetylase(s) that modifies H3K56. It is interesting that transcription factor binding and H3K56 acetylation generally do not occur at the exact same genomic location. This probably reflects the fact that the transcription factors we have examined cannot bind to DNA that is wrapped around the histone core. In contrast, E2F4 and C/EBPα can be found in much closer proximity to each other.

Genes that are bound by one or more factors or harbor the particular H3K56 acetylation modification are associated with higher expression levels, reflecting the general role of these transcription factors and histone modification in gene activation. Although H3K56 acetylation is widespread in human adipocytes, the genes that have the highest levels of acetylation are enriched for particular functions, including transcriptional regulation and adipokines and diabetes-related pathways. Many of the transcription factors with high levels of H3K56 acetylation have not been associated with adipocytes before. It is worth investigating the role of these transcription factors in adipocyte function.

Mikkelsen *et al.* recently profiled the genome-wide binding sites of PPARγ, CTCF and six histone modifications, namely H3K4me1, H3K4me2, H3K4me3, H3K27me3, H3K27ac and H3K36me3 in human adipose stromal cells (hASCs) derived from adult subcutaneous lipoaspirates [21]. We noticed that our H3K56 acetylation profile closely mirrors the open chromatin marks H3K4me2 and H3K27ac that they profiled, with 90% and 87% overlap, respectively. H3K56 acetylation has the least overlap (8%) with H3K27me3, which represents polycomb-mediated repression. Interestingly, the overlap of H3K56 acetylation with promoter initiation mark H3K4me3 is a moderate 55%, implying that H3K56 acetylation is more of an open chromatin mark rather than a transcription initiation mark.

Regarding the physiological role of H3K56 acetylation, recent work in Sirt6 knock-out mice reveals the association between hyperacetylation of H3K56 and obesity [16]: neural specific Sirt6-deleted mice have adult-onset obesity, and they show hyperacetylation at H3K9 and H3K56 in various brain regions [16]. The extent of H3K56 hyperacetylation is particularly widespread; it was observed in whole cell extracts from hypothalamus, cortex, hippocampus and cerebellum as well as purified whole brain nuclei. The authors suggested that the hyperacetylation observed may be related to a disruption of neuroendocrine regulation. In our study, we observed widespread H3K56 acetylation in human adipocytes; the degree of which is particularly significant in genes related to the adipokine signaling and Type II Diabetes Mellitus pathways. This gives rise to the possibility that H3K56, by means of its acetylation status, regulates diverse endocrine functions in both adipocytes and neurons. Profiling H3K56 and other histone acetylation sites in adipocytes in different metabolic conditions (for example, comparing insulin sensitive and insulin resistant adipocytes) and identifying the acetylase/deacetylases that are responsible for the modification will be an important step towards understanding the chromatin acetylation dynamics in normal and diseased states.

Our study is the first genome-wide H3K56 acetylation profile and binding location analysis for E2F4, HSF-1 and C/EBPα in human adipocytes. It provides a rich resource for future studies of the transcriptional regulation in human adipocytes.

## Materials and Methods

### Culturing human mesenchymal stem cell-derived adipocytes

Human mesenchymal stem cells (hMSC) at passage 2 (PT2501) were purchased from Lonza (Walkersville, MD), who obtained the cells from the bone marrow withdrawn from the posterior iliac crest of the pelvic bone of normal volunteers. Cells were plated in T-175 flasks at a density of 5000 cells/cm^2^ in MSC growth media (MSC-GM). For expansion of hMSC, cells were grown in T-175 flasks but prior to reaching confluence, cells were detached by trypsinization and replated at a density of 5000 cells/cm^2^. This process was repeated until the cells reach passage 4 to 7, when we induced the cells to differentiate: upon reaching confluence, the MSC-GM was removed and fresh adipogenic differentiation media (ADM; PT3004) containing 120 pM insulin, dexamethasone, IBMX, and indomethacin was added. The cultures were maintained in ADM for 14 days with media changes every 3–4 days. On day 14, the media was changed to adipogenic maintenance media (AMM) containing 120 pM insulin (PT4119A). On day 19, the adipocyte cultures were fixed by adding 20 ml serum free DMEM containing 1/10 volume of cross-linking solution (11% formaldehyde, 50 mM HEPES, 100 mM NaCl, 1 mM EDTA, 0.5 mM EGTA) for 10 minutes at room temperature. The formaldehyde was neutralized by the addition of 1 ml of 2.5 M glycine. Cultures were then rinsed twice in PBS (one quickly, one 5–10 minute) and then treated with 5 ml of Accutase solution (SCR005, Millipore) for 5 min at 37°C. Cells were then scraped and pooled into 50 ml tubes. Up to 30 T-175 flasks of cells were used for each adipocyte preparation.

### Human MSC-derived adipocytes purification

The adipocyte suspension from up to 10 T-175 flasks was pipetted up and down several times to enhance separation and then centrifuged for 25 minutes at 15°C at 3500 rpm. Due to their low density, the adipocytes floated at the surface while the denser non-adipocytes formed a pellet. The relatively pure adipocyte fraction formed a white layer (anti-pellet) at the meniscus and was transferred to a second tube by pipetting carefully. PBS was added to bring the volume to 50 ml and the tube was centrifuged for 5 minutes. The buoyant cell mass was harvested in the same manner, washed in PBS again and spun for 5 minutes. The PBS underneath the cells was carefully decanted and the cell mass (anti-pellet) was flash-frozen in liquid nitrogen. Each T-175 flask routinely yields ∼4 million cells, of which>50% (2 million) are adipocytes. All experiments were done using cells with passage number less than 8.

### Cellomics high content image analysis

Human bone marrow mesenchymal stem cells were induced to differentiate in 96-well plates as described above for 19 days and fixed with 4% paraformaldehyde solution for 30 minutes. The plates were washed with PBS, permeabilized with 0.1% Triton-X for 30 minutes and stained with PPARγ antibody (Cell Signaling Technologies, 10 µg/ml) overnight. Plates were washed with PBS, then DAPI (1 µg/ml), Nile red (1 µg/ml) and secondary Alexa-647 antibody (Invitrogen) were added and incubated for 2 hours at room temperature. Plates were washed with PBS, sealed and analyzed on a Cellomics Arrayscan. 100 fields per well of a 96-well plate were analyzed. DAPI, a nuclear stain, was used to count the number of nuclei per field. Nile red, a lipid droplet specific stain, was used to quantify the percent of differentiated adipocytes per field. Alexa 647 was used to quantify PPARγ expression and subcellular location.

### Chromatin Immunoprecipitation and high-throughput sequencing

Chromatin immunoprecipitation was done as described [17], with modifications. 5 ug of each antibody (listed in Table S2) was incubated with preblocked Dyanbead Protein A (Invitrogen 100.02D) overnight. The specificity of the antibodies was previously described [6], [12], [26], [27]. Human adipocytes were lysed in cold lysis buffer (20 mM HEPES, 0.25 M sucrose, 3 mM MgCl­_2_, 0.2% NP-40, 3 mM β-mercaptoethanol, 0.4 mM PMSF and protease inhibitor), dounced using a hand-held homogenizer for 20 strokes and incubated for 10 minutes at 4°C. Cells were then spun down and resuspended in ChIP SDS buffer (50 mM HEPES, 1% SDS, 10 mM EDTA and protease inhibitor). The cell lysate was sonicated using a Misonix Sonicator 3000 for 12 cycles; each cycle consisted of a 20-second pulse and a 1-minute pause. The sonicated chromatin was then diluted with ChIP dilution IP buffer (50 mM HEPES, 155 mM NaCl, 1.1% Triton-X, 0.11% sodium deoxycholate, 1 mM PMSF and protease inhibitor) and incubated with the antibody-coupled beads overnight. The beads were then washed five times with RIPA wash buffer and one time with TBS. The chromatin was eluted in ChIP elution buffer, reverse-crosslinked at 65°C overnight, treated with RNase and Proteinase K. The DNA was extracted with phenol-chloroform, precipitated with ethanol, purified and subjected to sequencing preparation using the Illumina sample preparation protocol. Sequencing was done using the Illumina Genome Analyzer II.

### ChIP-qPCR experiments

ChIP experiments for H3K56 acetylation, E2F4, HSF-1 and C/EBPα were performed as described above using separate adipocyte preparations. We chose 21 positive loci and 2 negative loci to verify the ChIP-seq peaks by qPCR. Enrichment was measured using SYBR Green I master mix in the Light Cycler 480 instrument (Roche) by comparing the percent input of the ChIP sample versus that of the IgG control sample, performed in triplicate using standard t-test. Only amplified products with a CP (crossing point) number of less than 33 were considered as positive. Primers sequences used for qPCR analysis were designed by oligo7, blasted against hg18 and a melt curve was generated for each reaction to make sure that only one product was amplified. Primer sequences are in Table S6.

### ChIP-seq data processing and peak calling algorithm

Unique sequences from each ChIP-seq experiment were extended 200 base pairs in a strand specific manner and allocated to 25-basepair bins across the genome. Peaks calling was done as described [28]: genomic bins containing statistically significant ChIP-seq enrichment were identified by comparison to a Poisson background model, using a p-value threshold of 10^−9^. The minimum number of counts in a genomic bin required for each sample to meet this threshold was calculated; they are 12, 8, 10 and 8 for H3K56 acetylation, C/EBPα, E2F4 and HSF-1 respectively. Bins that are within 200 bases of each other were combined to form regions. In order to take into account of the non-random nature of the background reads, an empirical background model was obtained from a mock IP experiment using IgG. We required that enriched regions have more than three-fold greater ChIP-seq density in the specific IP sample as compared with the non-specific IgG sample, normalized for the total number of reads. The raw data are deposited in GEO with accession number GSE24326.

### Gene expression analysis

Frozen cells were resuspended in Qiagen RLT buffer and total RNA isolated using the Qiagen RNeasy Kit. RNA was submitted to GeneLogic for cRNA preparation using the Affymetrix Genechip IVT Kit and hybridization to Affymetrix U133 Plus 2.0 microarrays. Expression raw data were processed using the Robust Multichip Average (RMA) R package in Bioconductor (http://bioconductor.org). During this processing, only the perfect match (PM) probe data were used; the mismatch (MM) probes were not used. The RMA processing consisted of background correction, quantile normalization to remove deleterious systematic effects between arrays and median polish used to estimate expression values and an additive model fit on log2(PM) values. The raw data are deposited in GEO with accession number GSE24422. All data is MIAME (Minimum Information about a Microarray Experiment) compliant.

### Gene ontology and pathway analysis

Gene Ontology analysis was done using PANTHER [29] and DAVID [30], [31]. Redundant GO categories were removed. All p-values presented were corrected for multiple hypothesis testing.

### Motif discovery method

Over-represented transcription factor motifs were identified in each experiment using THEME [19]. THEME uses principled statistical methods to test hypotheses about the binding specificity of the immunoprecipitated protein. Hypotheses are position weight matrix representations of DNA sequence motifs in the TRANSFAC and JASPAR database. They were evaluated based on their cross-validation error, which represents their ability to predict which sequences from a held-out test set were bound by the protein and which were not. Each hypothesis was either accepted or rejected based on a p-value, which was derived from comparing its predictive value to those of motifs derived by applying the same algorithm to randomly selected sequences for 25 times.

### Computation of correlation between ChIP-seq peaks

We identified regions of 1500 base pairs that contained binding sites peaks in the E2F4, C/EBPα and H3K56 acetylation data sets, where each region is centered around the E2F4 peak. For each enriched region, we computed three vectors, each represents the number of reads in one of the three datasets in 100-basepair bin, normalized by the total number of reads sequenced. These vectors were used to compute the correlation coefficient in figure 5B and are clustered and visualized as heatmaps in figure 5C.

## Supporting Information

Figure S1 **H3K56 acetylation enrichment is lower immediately before transcription start sites.** The top panel shows the normalized enrichment level of H3K56 acetylation with respect to transcription start sites from −10 kilobases to +10 kilobases in 100-basepair genomic bins. The normalized enrichment level in each genomic bin was computed by merging the reads across all genes, divided by the maximum number of reads in any genomic bin. The bottom panel is same as the top one with higher resolution around −1 kilobase to +1 kilobase with respect to transcription start sites.
(TIF)

Figure S2 **ChIP-qPCR verification of binding sites identified by ChIP-seq experiments.** Positive regions identified by ChIP-seq are indicated with *; positive regions verified by ChIP-qPCR are indicated with #. FP represents false positive and FN represents false negative. Y-axis shows percent of input and X-axis shows the particular ChIP experiment. (A) Selected H3K56 acetylation regions identified by ChIP-seq. (B) Selected bound regions identified by E2F4 ChIP-seq. (C) Selected bound regions identified by HSF-1 ChIP-seq. (D) Selected bound regions identified by C/EBPα ChIP-seq. (E) Selected bound regions identified by multiple ChIP-seq experiments. (F) Negative control.
(TIF)

Figure S3 **C/EBPα binds to adiponectin promoter and intronic enhancer.** Profile of C/EBPα binding sites along the adiponectin gene as shown on UCSC genome browser. Background represents the mock IgG experiment reads. Rectangular boxes below the C/EBPα peaks represent the enriched regions. The Y-axis represents the number of reads per million sequenced. The arrow indicates direction of transcription. Notice that there is a strong C/EBPα binding site upstream of the adiponectin transcription start site, a site that lies exactly at the transcription start site and another site (circle in red) that lies in the middle of the first intron, which has previously been identified as an intronic enhancer.
(TIF)

Table S1 **High content image analysis results of the differentiation efficiency of replicates of 6 MSC-derived adipocyte cultures.**
(XLSX)

Table S2 **Antibodies used in this study.**
(DOCX)

Table S3 **Number of reads, peaks and genes bound/enriched for each ChIP-seq experiment.**
(DOCX)

Table S4 **Number and percentage of genes with H3K56 acetylation and also bound by a particular transcription factor.**
(DOC)

Table S5 **List of genes that have H3K56 acetylation and also bound by at least one transcription factor.**
(XLSX)

Table S6 **Primer sequences used in this study.**
(XLSX)

Table S7 **Gene set enrichment results with a family-wise error rate of less than 0.05.**
(DOCX)

Table S8 **Gene ontology (GO) categories associated with the bound/enriched genes for each ChIP-seq experiment.**
(DOCX)
