# Supplementary figures and images for: Genome-Wide Profiling of H3K56 Acetylation and Transcription Factor Binding Sites in Human Adipocytes
Source: PLoS One. 2011 Jun 2;6(6):e19778. doi: 10.1371/journal.pone.0019778 (PMC3107206; doi:10.1371/journal.pone.0019778)

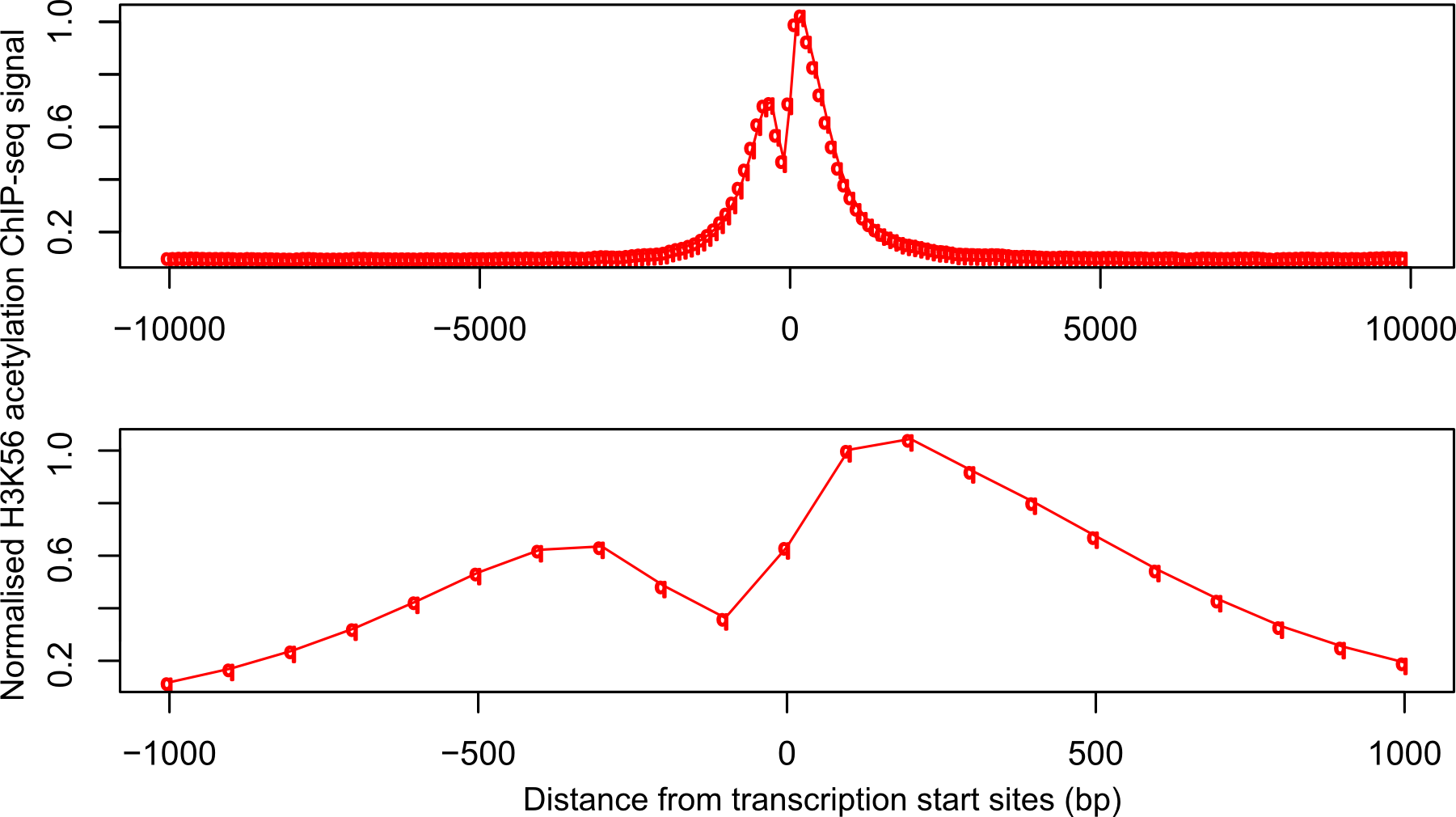

Supplement: Figure S1 — H3K56 acetylation enrichment is lower immediately before transcription start sites. The top panel shows the normalized enrichment level of H3K56 acetylation with respect to transcription start sites from −10 kilobases to +10 kilobases in 100-basepair genomic bins. The normalized enrichment level in each genomic bin was computed by merging the reads across all genes, divided by the maximum number of reads in any genomic bin. The bottom panel is same as the top one with higher resolution around −1 kilobase to +1 kilobase with respect to transcription start sites. (TIF) [file pone.0019778.s001.tif]

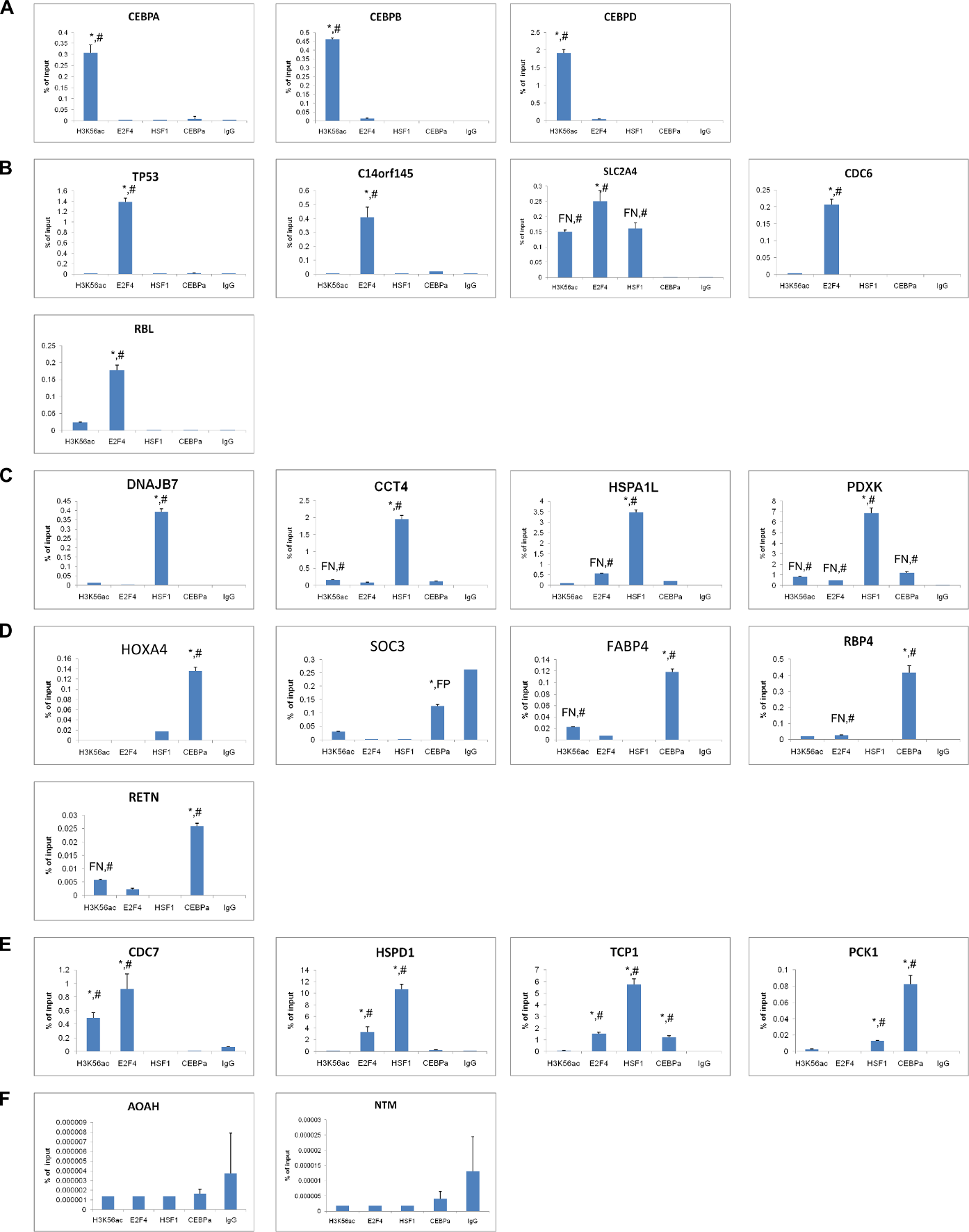

Supplement: Figure S2 — ChIP-qPCR verification of binding sites identified by ChIP-seq experiments. Positive regions identified by ChIP-seq are indicated with *; positive regions verified by ChIP-qPCR are indicated with #. FP represents false positive and FN represents false negative. Y-axis shows percent of input and X-axis shows the particular ChIP experiment. (A) Selected H3K56 acetylation regions identified by ChIP-seq. (B) Selected bound regions identified by E2F4 ChIP-seq. (C) Selected bound regions identified by HSF-1 ChIP-seq. (D) Selected bound regions identified by C/EBPα ChIP-seq. (E) Selected bound regions identified by multiple ChIP-seq experiments. (F) Negative control. (TIF) [file pone.0019778.s002.tif]

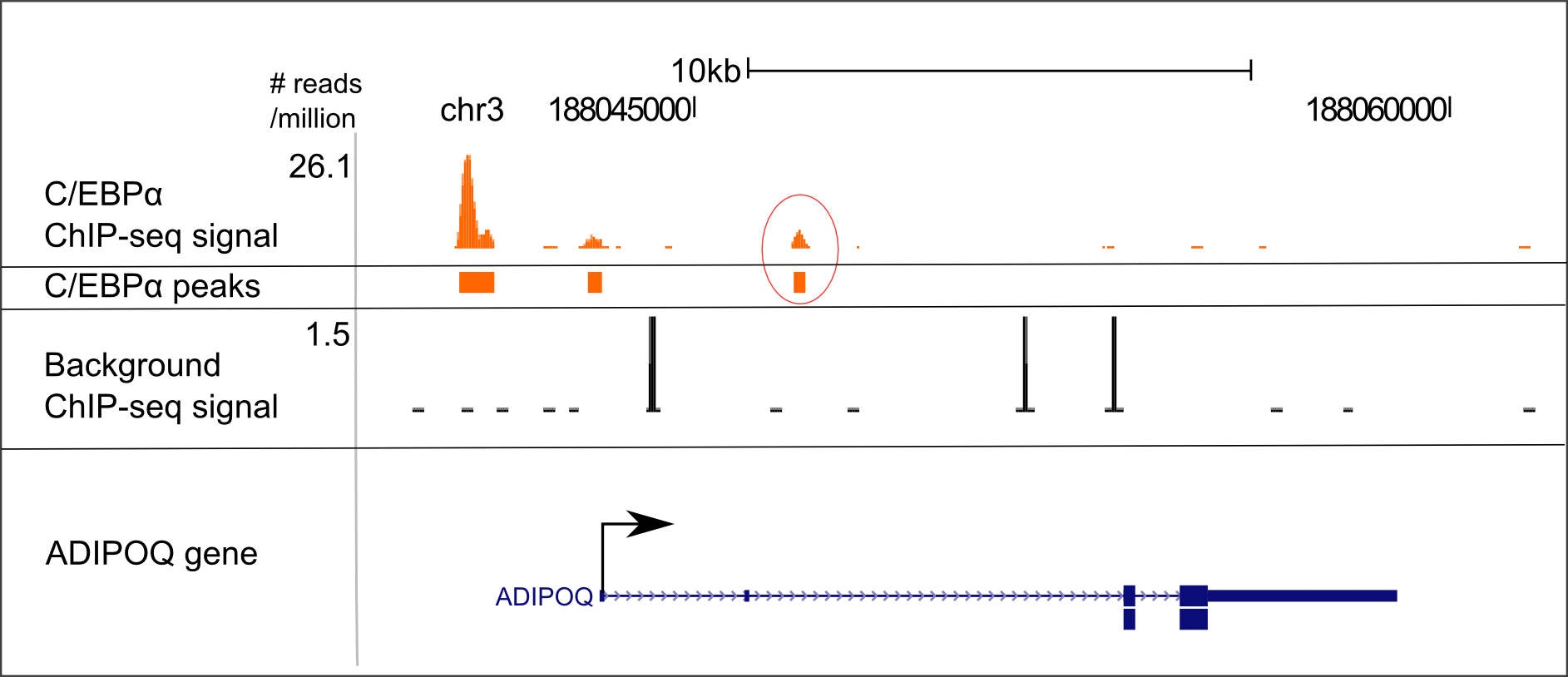

Supplement: Figure S3 — C/EBPα binds to adiponectin promoter and intronic enhancer. Profile of C/EBPα binding sites along the adiponectin gene as shown on UCSC genome browser. Background represents the mock IgG experiment reads. Rectangular boxes below the C/EBPα peaks represent the enriched regions. The Y-axis represents the number of reads per million sequenced. The arrow indicates direction of transcription. Notice that there is a strong C/EBPα binding site upstream of the adiponectin transcription start site, a site that lies exactly at the transcription start site and another site (circle in red) that lies in the middle of the first intron, which has previously been identified as an intronic enhancer. (TIF) [file pone.0019778.s003.tif]
