# Supplementary material for: Genome-Wide Profiling of H3K56 Acetylation and Transcription Factor Binding Sites in Human Adipocytes
Source: PLoS One. 2011 Jun 2;6(6):e19778. doi: 10.1371/journal.pone.0019778 (PMC3107206; doi:10.1371/journal.pone.0019778)
Supplement: Table S2 — Antibodies used in this study. (DOCX) [file pone.0019778.s005.docx]

**Table S2: Antibodies used for each experiment.**

| **Antibody** | **Vendor** | **Catalogue number** | **Lot number** |
| --- | --- | --- | --- |
| H3K56 acetylation | Epitomics | 2134-1 | YE101702C-2 |
| C/EBPα | Santa Cruz Biotechnology | sc-9314X | F1609 |
| E2F4 | Santa Cruz Biotechnology | sc-1082X | A2909 |
| HSF-1 | Santa Cruz Biotechnology | sc-9144 | L0407 |
| IgG | Santa Cruz Biotechnology | sc-2027 | J2909 |
