# Supplementary material for: Genome-Wide Profiling of H3K56 Acetylation and Transcription Factor Binding Sites in Human Adipocytes
Source: PLoS One. 2011 Jun 2;6(6):e19778. doi: 10.1371/journal.pone.0019778 (PMC3107206; doi:10.1371/journal.pone.0019778)
Supplement: Table S3 — Number of reads, peaks and genes bound/enriched for each ChIP-seq experiment. (DOCX) [file pone.0019778.s006.docx]

**Table S3: Number of reads sequenced, peaks and genes bound/enriched for each ChIP-seq experiment.**

| **Experiment** | **Number of reads** | **Number of peaks** | **Number of peaks in window*** | **Number of genes** |
| --- | --- | --- | --- | --- |
| H3K56 acetylation | 6,692,251 | 20,827 | 15,081 | 10,215 |
| C/EBPα | 3,222,053 | 32,864 | 6,853 | 5,818 |
| E2F4 | 4,102,521 | 6,193 | 4,658 | 5,340 |
| HSF-1 | 1,425,586 | 332 | 135 | 174 |

*window is defined as 10 kilobases upstream or downstream of transcription start sites.

The raw data are deposited in GEO with accession number GSE24326.
