# Supplementary material for: Genome-Wide Profiling of H3K56 Acetylation and Transcription Factor Binding Sites in Human Adipocytes
Source: PLoS One. 2011 Jun 2;6(6):e19778. doi: 10.1371/journal.pone.0019778 (PMC3107206; doi:10.1371/journal.pone.0019778)
Supplement: Table S4 — Number and percentage of genes with H3K56 acetylation and also bound by a particular transcription factor. (DOC) [file pone.0019778.s007.doc]

**Table S4: Number and percentage of genes with H3K56 acetylation that are also bound by a particular transcription factor**

|  | **Total #**  **of**  **bound genes** | **# of bound genes with H3K56 acetylation** | **% of bound genes with H3K56 acetylation** | **% of all acetylated genes that are bound** | **# of peaks with neighboring* H3K56 acetylation** | **% of peaks with neighboring* H3K56 acetylation** |
| --- | --- | --- | --- | --- | --- | --- |
| E2F4-  bound | 5340 | 4645 | 87.0% | 45.5% | 3444 | 73.9% |
| HSF-1-  bound | 174 | 157 | 90.2% | 1.5% | 93 | 68.9% |
| C/EBPα-  bound | 5818 | 4290 | 73.7% | 42.0% | 2108 | 30.7% |

*neighboring H3K56 acetylation means H3K56 acetylation occurs within 300 bases of the particular transcription factor bound site identified by ChIP-seq experiments.
