# Supplementary material for: Genome-Wide Profiling of H3K56 Acetylation and Transcription Factor Binding Sites in Human Adipocytes
Source: PLoS One. 2011 Jun 2;6(6):e19778. doi: 10.1371/journal.pone.0019778 (PMC3107206; doi:10.1371/journal.pone.0019778)
Supplement: Table S7 — Gene set enrichment results with a family-wise error rate of less than 0.05. (DOCX) [file pone.0019778.s010.docx]

**Table S7: Gene sets that show a positive association with the ranked H3K56 acetylated genes. Only results with a family-wise error rate (FWER) p-value < 0.05 are shown.**

| **Gene Set** | **N** | **NES** | **FWER p-value** |
| --- | --- | --- | --- |
| Cell Communication | 28 | 2.18 | 0.001 |
| Focal Adhesion | 109 | 2.05 | 0.011 |
| Adipocytokine Signaling Pathway | 47 | 2.01 | 0.018 |
| Type II Diabetes Mellitus | 20 | 1.95 | 0.03 |
| Melanoma | 38 | 1.93 | 0.04 |
| Bladder Cancer | 29 | 1.92 | 0.043 |

N: number of genes in set. NES: normalized enrichment statistics.
