# Supplementary material for: Genome-Wide Profiling of H3K56 Acetylation and Transcription Factor Binding Sites in Human Adipocytes
Source: PLoS One. 2011 Jun 2;6(6):e19778. doi: 10.1371/journal.pone.0019778 (PMC3107206; doi:10.1371/journal.pone.0019778)
Supplement: Table S8 — Gene ontology (GO) categories associated with the bound/enriched genes for each ChIP-seq experiment. (DOCX) [file pone.0019778.s011.docx]

**Table S8: Gene ontology (GO) categories associated with the bound/enriched genes for each ChIP-seq experiment.**

| **Biological Process (C/EBPα)** | **All** | **Found** | **Expected** | **p-value** |
| --- | --- | --- | --- | --- |
| Lipid, fatty acid and steroid metabolism | 717 | 247 | 187.8 | 4.26E-04 |
| Amino acid metabolism | 209 | 81 | 54.74 | 1.53E-02 |
| Other metabolism | 537 | 178 | 140.65 | 3.56E-02 |

| **Biological Process (H3K56ac)** | **All** | **Found** | **Expected** | **p-value** |
| --- | --- | --- | --- | --- |
| Nucleoside, nucleotide and nucleic acid metabolism | 2894 | 1925 | 1603.48 | 1.49E-16 |
| Protein metabolism and modification | 2518 | 1636 | 1395.15 | 1.47E-10 |
| Protein biosynthesis | 309 | 268 | 171.21 | 4.40E-10 |
| Intracellular protein traffic | 906 | 642 | 501.99 | 1.18E-08 |
| Protein modification | 1089 | 712 | 603.38 | 6.65E-04 |
| DNA metabolism | 297 | 224 | 164.56 | 7.52E-04 |
| Cell cycle | 866 | 569 | 479.82 | 7.99E-04 |
| Pre-mRNA processing | 252 | 193 | 139.63 | 1.37E-03 |
| mRNA splicing | 184 | 146 | 101.95 | 4.26E-03 |
| mRNA transcription | 1694 | 1055 | 938.59 | 6.43E-03 |
| Protein folding | 144 | 116 | 79.79 | 1.14E-02 |
| General vesicle transport | 249 | 183 | 137.96 | 1.88E-02 |
| Other metabolism | 537 | 351 | 297.54 | 3.61E-02 |

| **Biological Process (E2F4)** | **All** | **Found** | **Expected** | **p-value** |
| --- | --- | --- | --- | --- |
| Nucleoside, nucleotide and nucleic acid metabolism | 2894 | 1136 | 835.89 | 1.79E-26 |
| Cell cycle | 866 | 389 | 250.13 | 1.11E-15 |
| DNA metabolism | 297 | 170 | 85.78 | 5.10E-14 |
| Chromatin packaging and remodeling | 193 | 111 | 55.75 | 4.95E-09 |
| Pre-mRNA processing | 252 | 131 | 72.79 | 5.54E-08 |
| mRNA splicing | 184 | 103 | 53.15 | 1.38E-07 |
| DNA replication | 108 | 68 | 31.19 | 1.03E-06 |
| DNA repair | 158 | 91 | 45.64 | 3.51E-07 |
| Mitosis | 306 | 137 | 88.38 | 1.14E-04 |
| Protein biosynthesis | 309 | 137 | 89.25 | 1.88E-04 |
| Nuclear transport | 81 | 46 | 23.4 | 3.23E-03 |
| Cell cycle control | 397 | 161 | 114.67 | 2.98E-03 |
| Intracellular protein traffic | 906 | 316 | 261.69 | 1.37E-02 |
| DNA recombination | 38 | 25 | 10.98 | 3.74E-02 |
| Protein metabolism and modification | 2518 | 803 | 727.29 | 4.44E-02 |

| **Biological Process (HSF-1)** | **All** | **Found** | **Expected** | **p-value** |
| --- | --- | --- | --- | --- |
| Protein folding | 144 | 21 | 1.32 | 8.39E-17 |
| Protein metabolism and modification | 2518 | 54 | 23.13 | 2.19E-08 |
| Protein complex assembly | 65 | 8 | 0.6 | 2.93E-05 |
| Stress response | 191 | 11 | 1.75 | 2.77E-04 |
